# Effect of Cu valence states on conduction band position and reduction selectivity of TiO_2_-based heterojunction photocatalysts

**DOI:** 10.1016/j.isci.2025.112697

**Published:** 2025-05-19

**Authors:** Hong Qian, Binxia Yuan, Yuhao Liu, Li Wang, Rui Zhu, Pengyu Dong

**Affiliations:** 1 College of Energy and Mechanical Engineering, Shanghai University of Electric Power, Shanghai 201306, P.R. China; 2 Key Laboratory for Advanced Technology in Environmental Protection of Jiangsu Province, Yancheng Institute of Technology, Yancheng 224051, P.R. China

**Keywords:** Chemistry, catalysis

## Abstract

TiO_2_ has low photocatalytic activity due to its easy recombination of photogenerated charges and low visible light utilization efficiency. This study utilized the keto-enol tautomerism principle of fructose in an alkaline environment to successfully obtain Cu_2_O/TiO_2_ (CT), Cu_2_O/TiO_2_/Cu (CTC), and TiO_2_/Cu (TC) catalysts. As Cu(I) transformed into Cu(0), the bandgap narrowed, significantly enhancing visible light absorption. Photocatalytic tests showed that CT-0.15 exhibited the highest hydrogen production rate (279.53 μmol g^−1^ h^−1^), which was 25.18 times higher than that of TC-2. However, CTC-1 had the highest CO production rate (10.58 μmol g^−1^ h^−1^). Optoelectronic test revealed that CT-0.15 and CTC-1 had higher separation efficiency of photogenerated exciton. Density functional theory (DFT) calculations indicated that the change in the valence state of Cu influenced the reaction mechanism, with CT being favorable for the adsorption of H^+^ in HER and CTC promoting the adsorption of CO_2_ in carbon dioxide reduction reaction (CO2RR).

## Highlights

• Cu_2_O/TiO_2_, Cu_2_O/TiO_2_/Cu, and TiO_2_/Cu heterojunctions are successfully obtained
• Cu_2_O/TiO_2_ exhibits the highest H_2_ production rate of 279.53 μmol g^−1^ h^−1^
• The CO production of Cu_2_O/TiO_2_/Cu is higher that of than Cu_2_O/TiO_2_, with 10.58 μmol g^−1^ h^−1^
• The conversion of Cu^+1^ to Cu affected the selectivity of photocatalytic reduction products

## Graphical abstract

**Figure:**
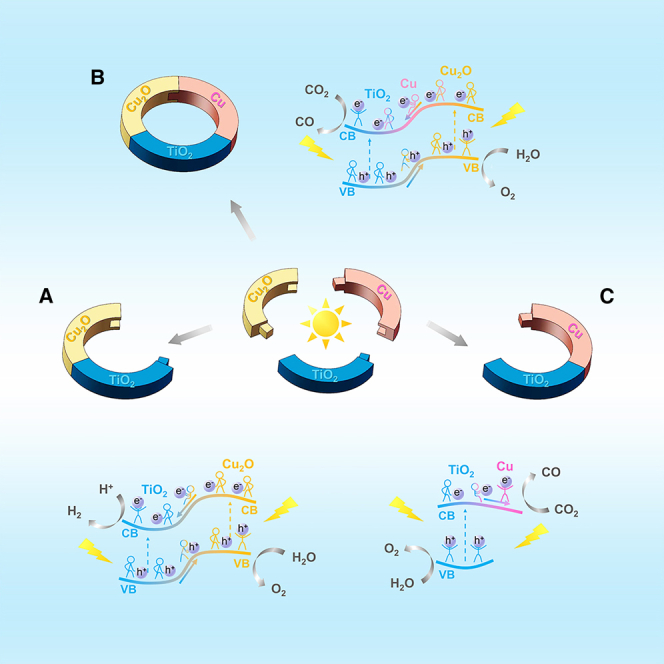

## Introduction

Photocatalysis has become an important means to alleviate global energy shortages and the greenhouse effect.^1^ TiO_2_ has become one of the candidate materials for photocatalysts due to its non-toxicity, chemical stability, and ease of preparation. However, the large bandgap of TiO_2_ (3.2 eV) leads to easy recombination of photogenerated charges and low utilization of visible light, resulting in low photocatalytic activity. To address this issue, various strategies, such as ion doping,^2^ noble metal deposition,^3^^,^^4^ morphology control,^5^^,^^6^ and heterostructure construction,^7^^,^^8^ have been employed to improve its photocatalytic activity. Particularly, heterojunctions have attracted widespread attention from researchers because they can rapidly separate and transfer photogenerated carriers, broaden the visible light response range, and alter the band positions to enhance catalytic activity.^9^^,^^10^^,^^11^^,^^12^

Currently, constructed heterojunctions, such as TiO_2_/Cu_2_O,^13^^,^^14^^,^^15^ TiO_2_/ZnO,^16^ TiO_2_/NiO,^17^ and TiO_2_/g-C_3_N_4_,^18^ have demonstrated higher photocatalytic activity than single TiO_2_ photocatalysts. However, the formation of heterostructures accelerates the transfer and accumulation of photogenerated charges, resulting in a lower rate of photocatalytic H_2_ production compared to the charge separation rate, leading to the accumulation of a large number of photogenerated electrons and holes.^19^^,^^20^ Cu_2_O tends to self-reduce and reduce photocatalytic activity.^21^ Therefore, adding metal nanoparticles to construct heterojunctions and accelerate the transfer of photogenerated charges has also received great attention.^22^ Li et al.^23^ electro-deposited Cu_2_O films on the surface of TiO_2_ nanorods with unique Au particles, showing significantly better photoelectrochemical cathode performance in hydrogen production and CO_2_ reduction compared to pure Cu_2_O and TiO_2_/Cu_2_O. Li et al.^24^ used a simple impregnation-reduction method to assemble core-shell nanoparticles of Cu@Cu_2_O on TiO_2_ nanotube arrays, which exhibited higher catalytic performance than Cu_2_O/TiO_2_-NTA and TiO_2_-NTAs.

Although researchers have extensively studied the construction of heterojunctions using Cu and its oxides, due to the complexity of the reaction processes, there has been little exploration of the selectivity of photocatalytic hydrogen production and CO_2_ reduction products under the same simple preparation method with different Cu valence states. Therefore, adopting a simple method to regulate the composition of heterojunctions to obtain more efficient photocatalysts and promote their widespread application is of guiding significance.

In this study, different Cu valence state Cu_2_O/TiO_2_, Cu_2_O-TiO_2_/Cu, and TiO_2_/Cu heterojunction catalysts were prepared by a one-step hydrothermal method. Experiments demonstrated that changes in Cu affected the position of the conduction band in the heterojunction, thereby influencing the selectivity of photocatalytic reaction products. Finally, the mechanism of the photocatalytic reaction was explained through density functional theory (DFT) calculations, providing a new approach for the efficient preparation of heterojunction composite catalytic materials.

## Results and discussion

### Structure properties

**Figure 1:**
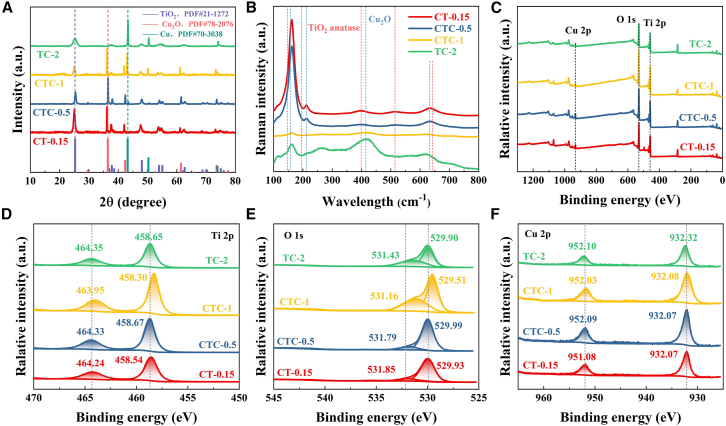
Valence information of CT-0.15, CTC-0.5, CTC-1, and TC-2 samples (A) XRD patterns. (B) Raman spectra. (C) XPS spectra of Survey. (D) XPS spectra of Ti 2p. (E) XPS spectra of O 1s. (F) XPS spectra of Cu 2p.

The X-ray diffraction (XRD) results (Figure 1A) showed that the characteristic peaks of anatase TiO_2_ (PDF#21–1272) were detected in all samples. The characteristic peaks of Cu_2_O were detected in CT-0.15, CTC-0.5, and CTC-1, and the diffraction peaks of Cu (PDF#70–3038) were detected in CTC-0.5, CTC-1, and CTC-2. As the amount of fructose increased, Cu(II) in copper acetate gradually converted to Cu(I) and Cu(0), and the TiO_2_ diffraction peaks gradually decreased and broadened. At 2θ = 25.3°, 36.4°, and 43.2°, compared to the standard PDF card, the positions of the diffraction peaks in the four samples shifted slightly. This was due to the mutual influence of the composition formation process and the lattice distortion at the interfaces in the one-step preparation method.

Figure 1B showed the Raman spectra of the CT-0.15, CTC-0.5, CTC-1, and TC-2 samples in the range of 100 cm^−1^ to 800 cm^−1^. Bending vibrations of Ti-O-Ti were observed at 146 cm^−1^, 196 cm^−1^, and 398 cm^−1^, while stretching vibrations of Ti-O-Ti were observed at 514 cm^−1^ and 641 cm^−1^. The Raman peaks at 146 cm^−1^, 196 cm^−1^, 398 cm^−1^, 514 cm^−1^, and 641 cm^−1^ represented the E_g_, E_g_, B_1g_, A_1g_, and A_1g_+B_1g_ vibrational modes of anatase TiO_2_, respectively.^25^^,^^26^ Among them, due to the partial overlap of the Cu_2_O mode, the Raman peaks of TiO_2_ became broader.^27^^,^^28^ The results indicated the presence of TiO_2_ and Cu_2_O in the heterojunction, which was consistent with the XRD results.

Figure 1C showed the X-ray photoelectron spectroscopy (XPS) spectra of CT-0.15, CTC-0.5, CTC-1, and TC-2. The presence of Cu, Ti, and O was observed. In Figure 1D, the Ti 2p XPS spectrum showed binding energies around 458 eV and 464 eV, corresponding to Ti 2p_3/2_ and Ti 2p_1/2_, respectively, indicating that Ti existed in the form of Ti^4+^ in the samples. The absence of a binding energy at 457 eV indicated that there was no Ti^3+^ 2p_3/2_. Figure 1E displayed the O 1s XPS spectrum, with binding energies around 530 eV and 532 eV corresponding to lattice oxygen and surface hydroxyl groups, respectively.^29^^,^^30^ Figure 1F showed the Cu 2p XPS spectrum, with binding energies at 932.07 eV and 952.09 eV corresponding to Cu 2p_3/2_ and Cu 2p_1/2_ of Cu_2_O, respectively, and binding energies at 932.32 eV and 952.10 eV corresponding to Cu 2p_3/2_ and Cu 2p_1/3_ of Cu, respectively.^21^^,^^22^^,^^23^^,^^24^^,^^25^^,^^26^^,^^27^^,^^28^^,^^29^^,^^30^^,^^31^^,^^32^^,^^33^^,^^34^ The binding energy shift in the Ti 2p and O 1s XPS spectra of CTC-1 indicated that the change from Cu(I) to Cu(0) increased the impact on the lattice distortion of TiO_2_. Additionally, the increase in Cu in the O 1s XPS spectrum showed that the proportion of hydroxyl groups in the samples increased, which was beneficial for enhancing photocatalytic activity.

**Figure 2:**
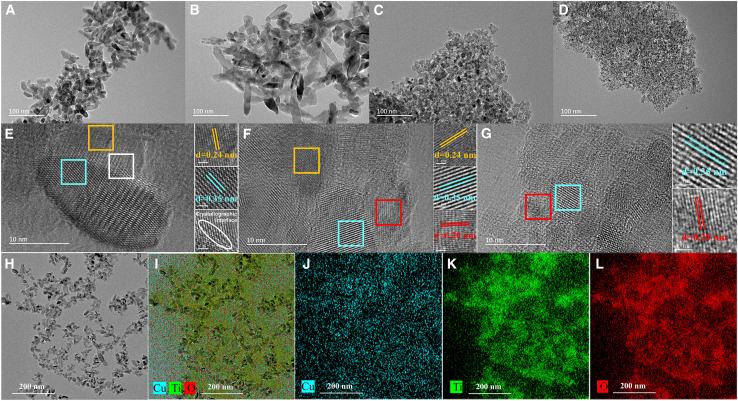
Morphology characterization (A) TEM image of CT-0.15 sample. (B) TEM image of CTC-0.5 sample. (C) TEM image of CTC-1 sample. (D) TEM image of TC-2 sample. (E) HRTEM image of CT-0.15 sample. (F) HRTEM image of CTC-1 sample. (G) HRTEM image of TC-2 sample. (H–L) EDX mapping of CT-0.15 sample.

Figures 2A–2D showed the transmission electron microscope (TEM) images of CT-0.15, CTC-0.5, CTC-1, and TC-2, respectively. With the increase in fructose content and changes in composition, the size of the samples gradually decreased. This might have been due to the increase in fructose accelerating the conversion of Cu(I) to Cu(0), leading to more nucleation and reduced growth. Meanwhile, the intermediate product of the reaction, gluconic acid, inhibited the growth of titanium dioxide. Figures 2E–2G displayed the high-resolution transition electron microscopy (HRTEM) images of CT-0.15, CTC-1, and TC-2, respectively. In Figure 2E, the lattice spacings of 0.24 nm belonging to the Cu_2_O(111) plane and 0.35 nm belonging to the TiO_2_(101) plane were measured, and small lattice distortions were present at the interface between the crystal planes. In the HRTEM images of CTC-1 and TC-2, the lattice spacings corresponding to the Cu_2_O(111) plane (0.24 nm), the TiO_2_(101) plane (0.35 nm), and the Cu(111) plane (0.20 nm) were measured, which was consistent with the XRD analysis. Figures 2H–2L showed the energy dispersive X-ray spectroscopy (EDX) maps of CT-0.15 with the uniform distribution of Cu, Ti, and O elements, indicating that the components of the prepared heterojunction catalysts were evenly distributed, which was beneficial for the transport of carriers within the heterojunction.

Copper acetate reacted with sodium hydroxide to form sodium acetate and copper hydroxide. Under alkaline conditions, fructose underwent keto-enol tautomerism to produce glucose. The ketone group in fructose was converted into the aldehyde group in glucose, which then reacted with copper hydroxide to produce gluconic acid and Cu_2_O. With the increasing fructose content in the reaction system, Cu(I) in Cu_2_O was further reduced to Cu(0). Meanwhile, tetrabutyl titanate hydrolyzed to produce n-butanol and TiO_2_. During this reaction, gluconic acid produced from the Cu_2_O reaction affected the hydrolysis of tetrabutyl titanate, and the n-butanol produced from the TiO_2_ reaction participated as a solvent in the formation of Cu_2_O. Cu_2_O and TiO_2_ nucleated and grew simultaneously, mutually promoting and constraining each other.

**Figure 3:**
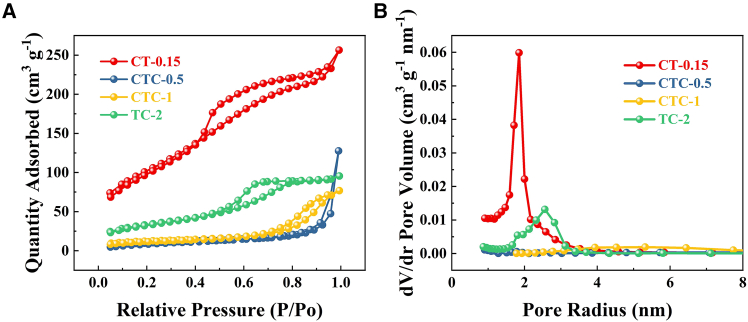
Analysis of different pores under N_2_ condition (A) Adsorption-desorption curves. (B) Pore size distribution.

To further analyze the microstructure of the samples, the N_2_ adsorption-desorption isotherms and pore size distributions of CT-0.15, CTC-0.5, CTC-1, and TC-2 samples were characterized, as shown in Figure 3. CT-0.15, CTC-0.5, CTC-1, and TC-2 exhibited type IV, V, V, and IV adsorption isotherms, respectively. CT-0.15 had an H4 hysteresis loop isotherm with no obvious saturation adsorption platform. Combined with TEM, CT-0.15 was found to be a solid with narrow slit-like pores, having a pore size of less than 2 nm, similar to activated carbon. TC-2 had an H_2_ hysteresis loop isotherm with a saturated adsorption platform. TEM revealed that TC-2 consisted of densely packed spherical particles with interstitial pores, with a pore size distribution between 2 and 3 nm. Therefore, CT-0.15 was mainly microporous, while TC-2 was primarily mesoporous. However, CT-0.5 and CTC-1 did not exhibit obvious microporous or mesoporous structures. The specific surface areas of CT-0.15, CTC-0.5, CTC-1, and TC-2 were calculated to be 370.362 m^2^/g, 35.082 m^2^/g, 41.510 m^2^/g, and 117.076 m^2^/g, respectively (see Table 1). A higher specific surface area indicated more active sites, which was beneficial for catalytic reactions. The higher specific surface areas of CT-0.15 and TC-2 were attributed to the pore structures present in the samples.

**Table 1:** Specific surface area, pore volume, and pore size of prepared samples

Sample	Surface Area (m^2^/g)	Pore volume (cm^3^/g)	Pore radius (nm)
CT-0.15	370.362	0.419	2.020
CTC-0.5	35.082	0.196	11.798
CTC-1	41.510	0.118	5.903
TC-2	117.076	0.147	2.349

### Photocatalytic properties

**Figure 4:**
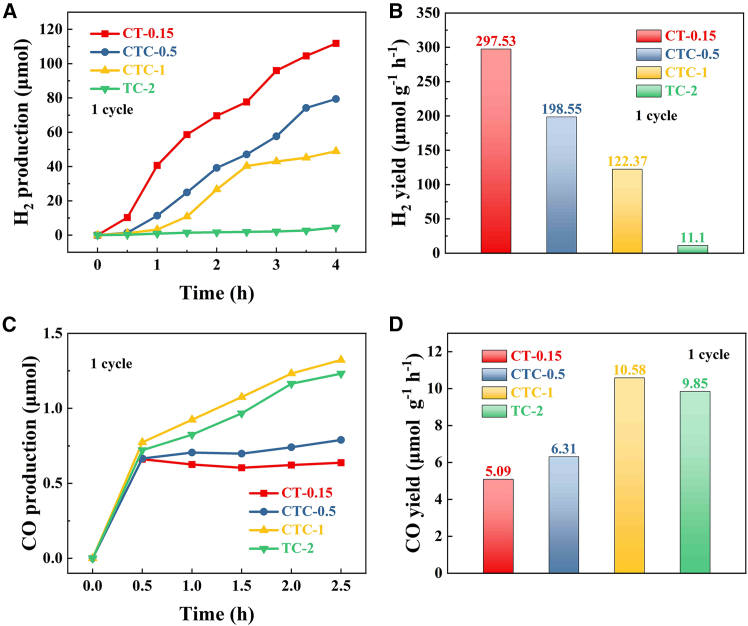
Photocatalytic properties of different samples (A) Photocatalytic H_2_ production. (B) Photocatalytic H_2_ yield. (C) Photocatalytic CO production. (D) Photocatalytic CO yield.

Figures 4A and 4B showed the H_2_ production and yield of CT-0.15, CTC-0.5, CTC-1, and TC-2 samples after 4 h of irradiation with a 300W xenon lamp. CT-0.15 started producing H_2_ early in the illumination period, and its H_2_ production was consistently much higher than that of CTC-0.5, CTC-1, and TC-2. The average hydrogen production rate of CT-0.15 in a single cycle reaction for 4 h is 279.53 μm mol g^−1^ h^−1^, which is 1.4 times, 2.3 times, and 25.2 times higher than CTC-0.5, CTC-1, and TC-2, respectively. To further investigate the relationship between sample composition and photocatalytic selectivity, the photocatalytic CO_2_ reduction performance of CT-0.15, CTC-0.5, CTC-1, and TC-2 samples was tested. The CO production and yield are shown in Figures 4C and 4D. During the tests, it was found that all samples exhibited high selectivity, with CO being the only reduction product. The average CO yield of CTC-1 in a single cycle reaction for 2.5 h is 10.58 μm mol g^−1^ h^−1^, which is 2.08 times, 1.68 times, and 1.07 times higher than CT-0.15, CTC-0.5, and TC-2, respectively. Compared with other studies, the catalyst prepared in this article has better catalytic performance (Table 2). Meanwhile, comparing CT-0.15, CTC-1, and TC-2, it was found that the change from Cu(I) to Cu(0) favored the shift of photocatalytic reduction products from H_2_ to CO. Comparing CTC-0.5 and CTC-1, it was found that an increase in the proportion of Cu(0) under the same composition conditions was more conducive to the high-selectivity reduction of CO_2_ to CO.

**Table 2:** Comparison of photocatalytic performance with literature

literature	Catalyst	Reaction conditions	Photocatalytic yield (μmol g^−1^ h^−1^)
Guan et al.^35^	BP/TiO_2_	Simulated sunlight (AM1.5)	H_2_: 112
Li et al.^36^	TiO_2_/rGO-EDA	UV light irradiation, ethanol solution	H_2_: 224.9
Yang et al.^37^	TiO_2_(A-R)/In_2_O_3_	300 W Xe lamp irradiation, 20% methanol as sacrificial agent	H_2_: 268
Dong et al.^38^	Co-MOF/Cu_2_O	Visible light irradiation, no solvent, 30°C water bath	CO: 3.83
Zhao et al.^39^	Ti_3_C_2_/g-C_3_N_4_	Visible light irradiation (λ ≥ 420 nm), no sacrificial agent	CO: 5.19 CH_4_: 0.044
Tang et al.^40^	UiO-66-NH_2_/Cu_2_O/Cu	20°C, 100 mL H_2_O, UV-Vis light irradiation (300 W Xe lamp)	CO: 4.54
This Work	Cu_2_O/TiO_2_ Cu_2_O/TiO_2_/Cu	20°C, 100 mL H_2_O, UV-Vis light irradiation (300 W Xe lamp)	H_2_:297.53 CO: 10.58

### Photoelectric properties

**Figure 5:**
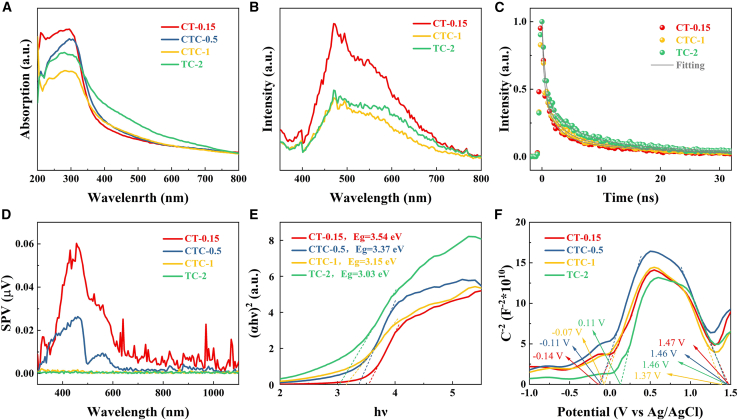
Photoelectric properties of different samples (A) UV–vis absorption spectra. (B) Steady-state PL. (C) Time-correlated PL. (D) SPV spectra. (E) Band gap evaluation from the plots of ${\left(\alpha hv\right)}^{2}$ versus hv. (F) Mott-Schottky curves.

To further analyze the catalytic selectivity of the heterojunctions with different components, their photoelectrochemical properties were characterized. The UV visible (UV-Vis) absorption spectra of CT-0.15, CTC-0.5, CTC-1, and TC-2 were shown in Figure 5A. Compared to the common TiO_2_, which could only absorb ultraviolet light with wavelengths shorter than approximately 385–400 nm, the absorption edges of all composite materials exhibited a redshift. Particularly, the TC-2 sample still demonstrated a high response within the visible light range, indicating that the construction of the heterojunction enhanced the visible light response of the catalytic materials, thereby improving their catalytic performance. CT-0.15 exhibited the strongest absorption intensity in both the UV and visible light regions, resulting in the highest hydrogen production efficiency among the samples. The TC-2 sample, with the highest Cu(0) content, showed a significant increase in visible light absorption intensity, indicating that the increase in metal elements broadened the light absorption range of TiO_2_.

Figure S1A displayed the electrochemical impedance spectroscopy (EIS) used to study the charge transfer impedance between the samples and the electrolyte. The smaller the diameter of the semicircle in the spectral curves, the smaller the charge transfer resistance of the sample. Thus, CT-0.15 exhibited the lowest impedance, indicating a faster electron transfer rate. The generation, migration, and recombination of photogenerated electrons and holes significantly impacted the catalytic performance of the heterojunctions. In Figure S1B, CT-0.15 exhibited a higher transient photocurrent density, indicating that the photogenerated electrons and holes in the sample could quickly separate, which was consistent with the EIS results and photocatalytic hydrogen production performance. In the initial stage of irradiation, significant shark peaks appeared due to uneven electron transport, leading to recombination on the electrode surface.^41^ Meanwhile, CTC-0.5 and CTC-1 exhibited higher photocurrent densities than TC-2, indicating that the surface plasmon resonance (SPR) effect of Cu was more pronounced in the multicomponent heterojunctions.^42^

Steady-state and transient photoluminescence (PL and TRPL) characterizations were used to further study the recombination and separation of photogenerated charges and holes in the heterojunctions. As shown in Figure 5B, the CTC-1 heterojunction exhibited significant fluorescence quenching, indicating that CTC-1 had good interfacial charge transfer capability, accelerating the separation and transfer of photogenerated electrons and holes.^43^^,^^44^ Additionally, the smoothness of the curves indicated low PL fluorescence intensity, suggesting that samples with different components had good interfacial charge transfer capabilities. Using a multi-exponential function equation to fit the corresponding TRPL curves (Equation 1), the average fluorescence lifetimes of CT-0.15, CTC-1, and TC-2 were calculated to be 3.38 ns, 3.93 ns, and 4.99 ns, respectively (Table S1).^45^ The fluorescence decay rate of CT-0.15 was higher than that of CTC-1 and TC-2, indicating rapid charge transfer at the CT-0.15 interface. Overall, the obtained heterojunction structures exhibited excellent charge separation and rapid transfer capabilities.

(Equation 1) $$y=A_{1}\cdot e^{\left(\frac{-x}{\tau_{1}}\right)}+A_{2}\cdot e^{\left(\frac{-x}{\tau_{2}}\right)}+A_{3}\cdot e^{\left(\frac{-x}{\tau_{3}}\right)}+y_{0}$$

To study the transmission path of charge carriers on the catalyst surface, the photoelectric properties of CT-0.15, CTC-0.5, CTC-1, and TC-2 were studied using surface photovoltage (SPV), as shown in Figure 5D. A positive SPV response indicated that photogenerated holes moved to the catalyst surface, while photogenerated electrons moved to the surface in the opposite direction.^46^ Characterization revealed that the SPV responses of the samples were positive, with the response intensity of CT-0.15 being significantly higher than those of CTC-0.5, CTC-1, and TC-2, indicating a large number of holes generated on the CT-0.15 surface. Additionally, the SPV spectra in the visible light range were similar to the absorption spectra, indicating that photoexcitation induced the generation and separation of photogenerated charges.^47^^,^^48^ The SPV peak values gradually decreased with the transition from Cu(I) to Cu(0), suggesting that electron transmission in the heterojunctions became more pronounced.

**Figure 6:**
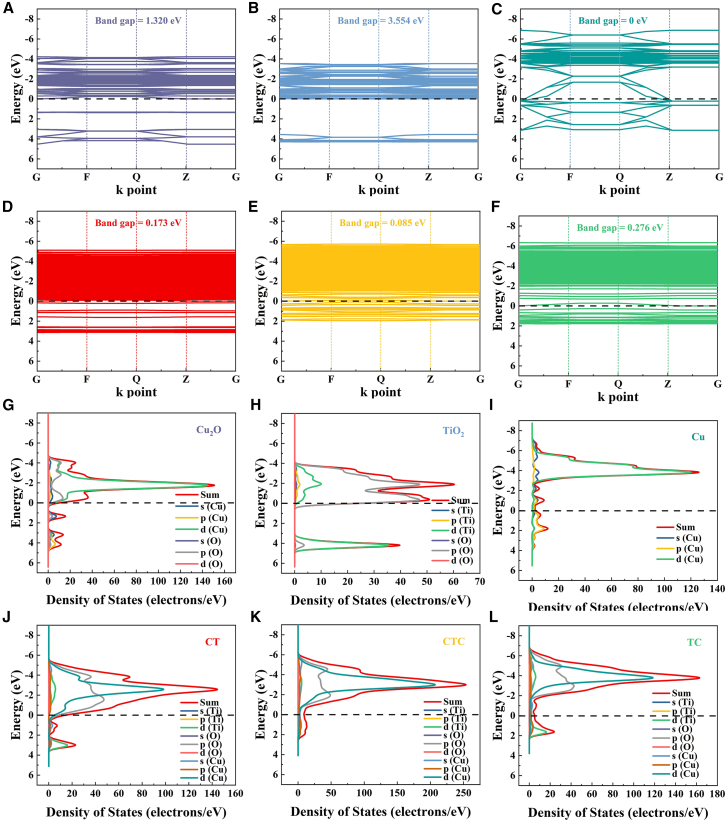
DFT calculation of energy bands and density of states for different models (A and G) Cu_2_O(111) facet model. (B and H) TiO_2_(101) facet model. (C and I) Cu(111) facet model. (D and J) CT model. (E and K) CTC model. (F and L) TC model.

Figure 5E showed the bandgap diagrams of the UV-Vis absorption spectra calculated using the Kubelka-Munk equation.^49^ The bandgaps of CT-0.15, CTC-0.5, CTC-1, and TC-2 were 3.54 eV, 3.37 eV, 3.15 eV, and 3.03 eV, respectively. The bandgaps of the samples gradually decreased with changes in composition. This was due to the surface plasmon resonance effect of metallic Cu promoting changes in the Fermi level.^50^ To determine the relationship between Cu valence changes and band edge positions in the heterojunctions and further elucidate the catalytic mechanism, Mott-Schottky curves were used for characterization. Typically, p-type semiconductors show a negative slope in the Mott-Schottky curve, while n-type semiconductors show a positive slope.^51^ As shown in Figure 5F, all heterojunction samples exhibited both positive and negative slopes, indicating the presence of p-type (Cu_2_O) and n-type (TiO_2_) semiconductors in the heterojunctions. Using the Mott-Schottky equation, the flat band potentials of CT-0.15, CTC-0.5, CTC-1, and TC-2 heterojunctions were determined, as shown in Table 3. Typically, compared to the flat band potential, the bottom of the conduction band (CB) of an n-type semiconductor will have a more negative potential of 0.3 eV, while the top of the valence band (VB) of a p-type semiconductor will have a more positive potential of 0.3 eV.^52^ Using the combined calculation formula, the bandgap widths of CT-0.15, CTC-0.5, CTC-1, and TC-2 were obtained as 2.21 eV, 2.17 eV, 2.13 eV, and 1.86 eV, respectively (Table 3). The change pattern of the bandgap width was consistent with Figure 5E. Additionally, as seen in Figure 5F, the transition from Cu(I) to Cu(0) had a more pronounced effect on the position of the conduction band. Therefore, the change in Cu valence directly affected the occurrence of reduction reactions on the conduction band side, promoting high selectivity of the reduction products.

**Table 3:** Flat-band potential, valence band, conduction band, and bandgap width obtained from the Mott-Schottky curves

Sample	E_fb_	E_CB_	E_fb_’	E_VB_	E_g_
CT-0.15	−0.14	−0.44	1.47	1.77	2.21
CTC-0.5	−0.11	−0.41	1.46	1.76	2.17
CTC-1	−0.07	−0.37	1.46	1.76	2.13
TC-2	0.11	−0.19	1.37	1.67	1.86

E_fb_ and E_fb_' in the table represent the flat band potential, which were the focal values of the positive and negative slopes of the Mott-Schottky curves and the *x* axis, respectively; E_CB_ was the conduction band potential, and E_VB_ was the valence band potential; E_g_ was the bandgap width.

### Theoretical calculation and mechanism analysis

Using Materials Studio software, constructed models for CT, CTC, TC, and their corresponding HER and carbon dioxide reduction reaction (CO2RR) models, as shown in Figure S2. The band structures and density of states (DOS) of Cu_2_O, TiO_2_, Cu, CT, CTC, and TC were obtained through DMol3 calculations (Figure 6). In the band structure diagrams, defect levels were observed in the heterojunction catalyst models, which facilitated electron transport. Comparing the band structures of CT, CTC, and TC, we found that the conduction band position shifted more negatively as the Cu(0) proportion increased in the samples, consistent with the Mott-Schottky measurements. Similarly, this trend was observed in the DOS diagrams of CT, CTC, and TC. As shown in Figures 6J–6L, the conduction band in the heterostructure was primarily contributed by the Cu3d orbitals, indicating that changes in Cu valence state affected the reduction reactions at the conduction band, enhancing the selectivity of the heterojunction catalysts.

**Figure 7:**
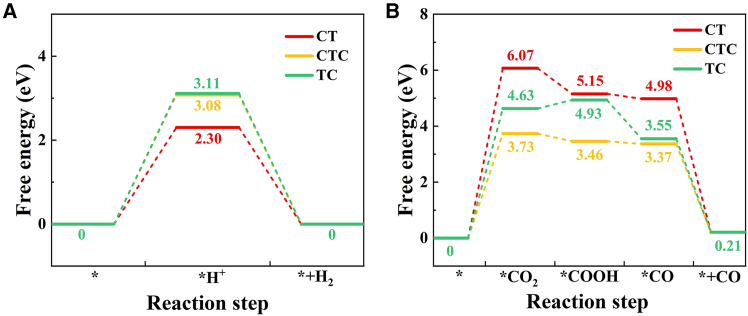
Free energy step diagrams of different models (A) HER. (B) CO2RR.

HER and CO2RR for the CT, CTC, and TC heterojunction models were calculated to directly evaluate the differences in catalytic performance, as shown in Figure 7. When a H^+^ was adsorbed on the surface of the heterojunction models, the Gibbs free energies of CT, CTC, and TC were 2.30 eV, 3.08 eV, and 3.11 eV, respectively. This indicated that the difficulty of the photocatalytic hydrogen evolution reaction increased, consistent with the H_2_ production rates shown in Figure 4. For CO2RR, the key step in the catalytic reaction for the CT, CTC, and TC heterojunctions occurred at the ∗CO_2_ step, with Gibbs free energies of 6.07 eV, 4.63 eV, and 3.73 eV, respectively, suggesting that CTC was more favorable for CO_2_ adsorption compared to CT and TC. This agreed with the catalytic performance test results in Figure 4, further demonstrating that heterojunctions with different Cu valence states had varying photocatalytic selectivity.

**Figure 8:**
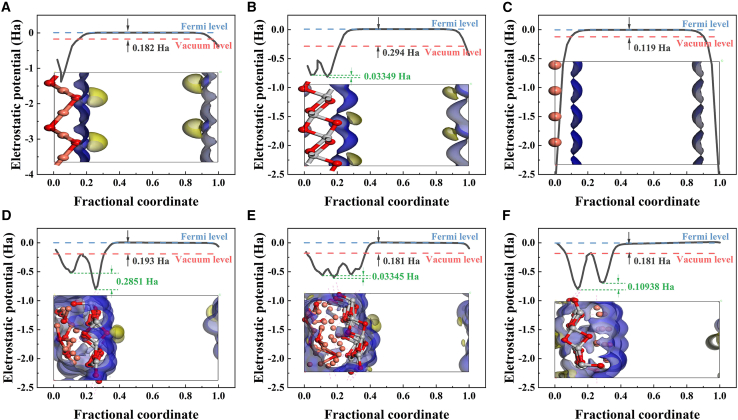
Optimized structural model and calculated work function (A) Cu_2_O (111) facet model. (B) TiO_2_(101) facet model. (C) Cu(111) facet model. (D) CT model. (E) CTC model. (F) TC model.

**Figure 9:**
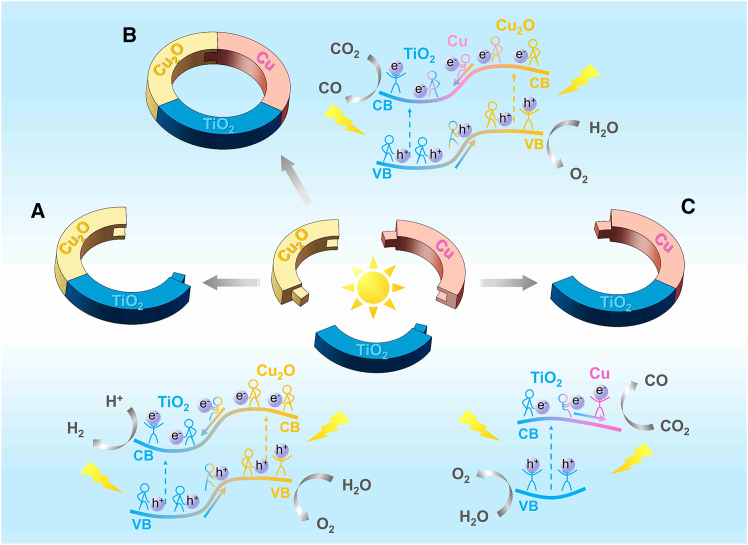
Photocatalytic reaction mechanism diagram (A) Cu_2_O/TiO_2_. (B) Cu_2_O/TiO_2_/Cu. (C) TiO_2_/Cu.

The work functions of Cu_2_O, TiO_2_, Cu, CT, CTC, and TC were calculated to further explain the differences in catalytic performance, as shown in Figure 8. The work function is the difference between the Fermi level and the vacuum level; the larger the work function, the easier it is for the material to become an electron acceptor, and conversely, it more readily becomes an electron donor. When Cu_2_O (0.182 Ha) was in contact with TiO_2_ (0.294 Ha), electrons in the conduction band of Cu_2_O more easily transferred to the conduction band of TiO_2_, and holes in the valence band of TiO_2_ more easily transferred to Cu_2_O, forming a typical type-II heterojunction (see Figure 9). Combined with the surface differential charge density (Figure S3), it was evident that in CT, electrons were gained on the TiO_2_ surface and lost on the Cu_2_O surface. In CTC, a significant positive peak appeared at Cu(0), indicating that Cu accelerated electron transfer.

In the work function diagrams of CT, CTC, and TC, the built-in electric field of CT was as high as 0.2851 Ha, which was 8.5 times and 2.6 times higher than that of CTC and TC, respectively, favoring the separation and transport of photogenerated electrons and holes, thus enhancing the efficiency of the photocatalyst. Additionally, using highly oriented pyrolytic graphite (HOPG) as the substrate, the surface potentials of CT-0.15, CTC-1, and TC-2 were measured, as shown in Figure S4. The work functions obtained by selecting potential points with high distribution probability in the images through Equation 2 ^53^ matched those obtained through DFT calculations, further confirming the reliability of the proposed electron transport pathways.

(Equation 2) $$\phi_{\text{sam}ple}=\phi_{HOPG}+e^{{Test}_{HOPG}-{Test}_{sample}}$$

### Conclusions

In summary, uniform Cu_2_O/TiO_2_, Cu_2_O/TiO_2_/Cu, and TiO_2_/Cu heterojunctions with different Cu valence states were prepared through a one-step hydrothermal method. As Cu(I) transitioned to Cu(0), the morphology and size of the samples gradually decreased, the bandgap of the heterojunction catalysts narrowed, and the visible light absorption range significantly broadened. EIS, transient photocurrent curves, PL, TRPL, and SPV indicated that CT-0.15 and CTC-1 had better separation efficiency of photogenerated electrons and holes. The PL intensity of CTC-1 was half that of CT-0.15, and compared to TC-2, the average fluorescence lifetime of CTC-0.5 was reduced by one-third. The SPV peak value was six times that of TC-2. After 4 h of visible light exposure, the average hydrogen production rate of CT-0.15 was 279.53 μmol g^−1^ h^−1^, 2.3 times that of CTC-1. However, the average CO production rate of CTC-1 for CO_2_ reduction was 10.58 μmol g^−1^ h^−1^, 2.08 times that of CT-0.15, 1.68 times that of CTC-0.5, and 1.07 times that of TC-2. Gibbs free energy step diagrams of HER and CO2RR showed that CT more easily adsorbed H^+^ compared to CTC and TC, with key steps in the photocatalytic reaction of the heterojunction catalysts occurring in the ∗CO_2_ step, and CTC required less energy (3.73 eV). Combined with the flat band potentials from M-S curves, surface potentials from SPM, work functions from DFT calculations, and differential charge density properties, it was shown that changes in Cu valence states directly affected the reduction reactions on the conduction band side of the heterojunction catalysts. Finally, the possible formation reasons for changes in Cu valence states during catalyst preparation were analyzed, providing insights for the further preparation of highly selective heterojunction catalysts.

### Limitations of the study

In this study, we prepared uniform Cu_2_O/TiO_2_, Cu_2_O/TiO_2_/Cu, and TiO_2_/Cu heterojunctions with different valence states of Cu through a one-step hydrothermal method. The experimental results showed that the heterojunctions formed between Cu in different valence states and TiO_2_ exhibited different catalytic activities in photocatalytic hydrogen production and photocatalytic CO_2_ reduction. Since the prepared composites were heterostructures, it was difficult to accurately determine the band gap, conduction band, and valence band positions of a single component within the heterojunction, which limited the discussion of the catalytic reaction mechanism. In the future, we will continue to use more advanced techniques to further investigate the related systems and gain a deeper understanding of the underlying mechanisms.

## Resource availability

### Lead contact

Further information and requests for resources should be directed to the lead contact, Binxia Yuan (yuanbinxia100@163.com).

### Materials availability

This study did not generate new materials.

### Data and code availability

• Any additional information required to reanalyze the data reported in this paper is available from the lead contact upon request.
• Data Availability Statement: All data reported in this paper will be shared by the lead contact upon request.
• Code: This paper does not report original code.

## Acknowledgments

This work was supported by the financial supports from 10.13039/501100001809National Natural Science Foundation of China (no. 12172210 and 21403184).

## Author contributions

Conceptualization, H.Q. and B.Y.; writing the original draft, H.Q.; methodology, H.Q. and Y.L.; writing-review and editing, B.Y., W.L., and P.D.; funding acquisition, R.Z.

## Declaration of interests

The authors declare no competing interests.

## STAR★Methods

### Key resources table

**Table:** 

REAGENT or RESOURCE	SOURCE	IDENTIFIER
**Chemicals, peptides, and recombinant proteins**		

copper acetate	Macklin	6064-93-1
fructose	Macklin	7660-25-5
tetrabutyl titanate	Macklin	5593-70-4
potassium ferricyanide	Macklin	13746-66-2
ethanol	Macklin	64-17-5
sodium hydroxide	Aladdin	1310-73-2
sodium sulfate	Shanghai Chemical Reagent	7757-82-6

### Method details

#### Materials

All chemicals were used directly without further purification. Copper acetate (Cu(CH_3_COO)_2_, AR, 99.0%), fructose (C_6_H_12_O_6_, 99%), titanium butoxide (C_16_H_36_O_4_Ti, analytical reagent), potassium ferricyanide (K_3_[FeC_6_N_6_], AR, 99.5%), and ethanol (C_2_H_5_OH, 99.7%) were purchased from Macklin. Sodium hydroxide (NaOH, GR, 97%) was purchased from Aladdin. Sodium sulfate (Na_2_SO_4_, AR) was purchased from Shanghai Chemical Reagent.

#### Preparation of Cu_2_O/TiO_2_, Cu_2_O/TiO_2_/Cu and TiO_2_/Cu

In a typical procedure, 0.5 mmol Cu(CH_3_COO)_2_, an amount of fructose, and NaOH were added into 50 mL distilled water, and 0.5 mL titanium butoxide was dripped into the above suspension under stirred at room temperature for 30 min. Then, the mixed solution was transferred into a 100 mL Teflon-lined stainless-steel autoclave, heated at 180°C for 4 h, and cooled down naturally. After that, the resulting solid products were collected by centrifugation, washed several times with ethanol and distilled water, then dried at 60°C for 12 h. According to the different fructose content and sample composition, the obtained samples were named CT-0.15, CTC-0.5, CTC-1, and TC-2, where the first “C” represented Cu_2_O, the second “T” represented TiO_2_, the third “C” represented Cu, and the number represented the molar of fructose (mmol).

#### Characterization of the catalyst

The samples were recorded on the Bruker D8 Advance X-ray diffractometer at 10° to 80° (2θ) X-ray diffraction (XRD) spectra within the range to evaluate the composition of the samples. The morphology was characterized by transmission electron microscopy (JEM-2100). Specific surface area and pore size were characterized by N_2_ absorption-desorption test (ASAP 2460). X-ray photoelectron spectroscopy (XPS) was obtained by Thermo Scientific K-Alpha. The absorption spectra of the samples were obtained by Shimadzu UV-3600 spectrophotometer. Steady-state surface photovoltage (SPV) was obtained by a surface photovoltage testing system (CEL-SPS1000).

#### Electrochemical measurement

The electrochemical measurement was operated on the CHI760E electrochemical workstation. In a typical three-electrode, Ag/AgCl electrode and Pt foil were used as reference electrode and counter electrode, respectively, and samples were dropped on 1 × 1.5 cm^2^ FTO conductive glass and dried to form films as the working electrode. 2.5 M K_3_[FeC_6_N_6_] electrolyte was used for electrochemical impedance spectroscopy (EIS) and Mott Schottky curves (MS) measurements and 0.1 M Na_2_SO_4_ was used for the transient photocurrent curve (I-t). A 500 W xenon lamp (CEL-S500-T5) equipped with AREF total reflection filter was used as the light source.

#### Photocatalytic performance

All photocatalytic H_2_ production was carried out in the photocatalytic system equipped with a 300 W Xe lamp (Labsolar-6A). A gas chromatograph (Fuli 97Plus) equipped with a TCD detector was used for the online separation and detection of H_2_. Added 100 mg photocatalyst to 100 mL water solution with a volume fraction of 10% methanol.

Photocatalytic CO_2_ reduction was tested in the photocatalytic reaction system (MC-SPH2O-A). Firstly, the photocatalyst (50 mg) was dispersed in distilled water (100 mL). Then, degassed and washed the mixture to about −0.1 MPa. Argon was used as the carrier gas in production, 300 W Xenon lamp was used as the light source, and the quantity of reduction products was quantitatively analyzed by GC2014C gas chromatography.

#### DFT calculation models and methods

In this work, calculations were completed using the DMol3 module in the first-principles Material Studio (MS) software based on density functional theory (DFT). The Perdew-Burke-Ernzerhof (PBE) method within the generalized gradient approximation was chosen as the calculation method. The anatase TiO_2_(101) crystal plane (space group 141 I41/AMD), Cu_2_O (111) crystal plane (space group 224 PN-3M), and Cu (111) crystal plane (space group 225 FM-3M) were cleaved. Heterojunction models of Cu_2_O(111)/TiO_2_(101), Cu_2_O(111)/TiO_2_(101)/Cu(111), and TiO_2_(101)/Cu(111) with a vacuum of 15 Å were established, and models of photocatalytic hydrogen production and CO_2_ reduction processes were constructed based on these three models. The DMol3 module was used to optimize the geometries of all models, with a k-point grid of 1×1×1 selected in the Brillouin zone. Full relaxation was performed for all atoms in the unit cell, with a relaxation convergence accuracy of 1.0×10^−6^ Ha, a maximum stress setting of 0.002 Ha/Å, and a maximum displacement not exceeding 0.5Å. The optimized models were used to calculate properties such as band structure, density of states, work function, and Gibbs free energy in the DMol3 module.

### Quantification and statistical analysis

XPS spectra quantification was carried out using the Casa XPS analysis software. Statistical analysis of data was performed using Origin (Origin Lab).

## References

[1] Nie N., Zhang L., Fu J., Cheng B., Yu J. (2018). Self-assembled hierarchical direct Z-scheme g-C_3_N_4_/ZnO microspheres with enhanced photocatalytic CO_2_ reduction performance. Appl. Surf. Sci..

[2] Huang J., Dou L., Li J., Zhong J., Li M., Wang T. (2021). Excellent visible light responsive photocatalytic behavior of N-doped TiO_2_ toward decontamination of organic pollutants. J. Hazard. Mater..

[3] Zhang Y., Liu J.X., Qian K., Jia A., Li D., Shi L., Hu J., Zhu J., Huang W. (2021). Structure Sensitivity of Au-TiO_2_ Strong Metal–Support Interactions. Angew. Chem. Int. Ed..

[4] Chen Y., Ji S., Sun W., Lei Y., Wang Q., Li A., Chen W., Zhou G., Zhang Z., Wang Y. (2020). Engineering the Atomic Interface with Single Platinum Atoms for Enhanced Photocatalytic Hydrogen Production. Angew. Chem. Int. Ed..

[5] Zheng Z., Huang B., Lu J., Wang Z., Qin X., Zhang X., Dai Y., Whangbo M.-H. (2012). Hydrogenated titania: synergy of surface modification and morphology improvement for enhanced photocatalytic activity. Chem. Commun..

[6] Cheng Q., Yuan Y.-J., Tang R., Liu Q.-Y., Bao L., Wang P., Zhong J., Zhao Z., Yu Z.-T., Zou Z. (2022). Rapid Hydroxyl Radical Generation on (001)-Facet-Exposed Ultrathin Anatase TiO_2_ Nanosheets for Enhanced Photocatalytic Lignocellulose-to-H_2_ Conversion. ACS Catal..

[7] Hieu V.Q., Phung T.K., Nguyen T.-Q., Khan A., Doan V.D., Tran V.A., Le V.T. (2021). Photocatalytic degradation of methyl orange dye by Ti_3_C_2_–TiO_2_ heterojunction under solar light. Chemosphere.

[8] Yan J., Wu H., Chen H., Zhang Y., Zhang F., Liu S.F. (2016). Fabrication of TiO_2_/C_3_N_4_ heterostructure for enhanced photocatalytic Z-scheme overall water splitting. Appl. Catal. B Environ..

[9] Akhundi A., Zaker Moshfegh A., Habibi-Yangjeh A., Sillanpää M. (2022). Simultaneous Dual-Functional Photocatalysis by g-C_3_N_4_ -Based Nanostructures. ACS ES. T. Eng..

[10] Hemmati-Eslamlu P., Habibi-Yangjeh A. (2024). A review on impressive Z- and S-scheme photocatalysts composed of g-C_3_N_4_ for detoxification of antibiotics. FlatChem.

[11] Habibi-Yangjeh A., Pournemati K. (2024). A review on emerging homojunction photocatalysts with impressive performances for wastewater detoxification. Crit. Rev. Environ. Sci. Technol..

[12] Li Y., Wu Z., Liu T., Song Z., Zhang Y. (2021). Modulating Photon Harvesting Through Constructing Oxygen Vacancies-Rich 0D/2D Plasmonic Bi/Bismuth Oxybromide Upconversion Nanosheets Toward Improved Solar Photocatalysis. Sol. RRL.

[13] Yang G., Qiu P., Xiong J., Zhu X., Cheng G. (2022). Facilely anchoring Cu_2_O nanoparticles on mesoporous TiO_2_ nanorods for enhanced photocatalytic CO_2_ reduction through efficient charge transfer. Chin. Chem. Lett..

[14] Aguirre M.E., Zhou R., Eugene A.J., Guzman M.I., Grela M.A. (2017). Cu_2_O/TiO_2_ heterostructures for CO_2_ reduction through a direct Z-scheme: Protecting Cu_2_O from photocorrosion. Appl. Catal. B Environ..

[15] Wang X., Qiao P., Chen Q., Dai M., Liu Y., Wang Y., Wang W., Liu Y., Song H. (2023). Electrochemically Deposited Cu2O-Doped TiO_2_ Nanotube Photoanodes for Hydrogen Evolution. Catal. Lett..

[16] Nguyen C.H., Tran M.L., Tran T.T.V., Juang R.-S. (2020). Enhanced removal of various dyes from aqueous solutions by UV and simulated solar photocatalysis over TiO_2_/ZnO/rGO composites. Separ. Purif. Technol..

[17] Zhao H., Li C.-F., Liu L.-Y., Palma B., Hu Z.-Y., Renneckar S., Larter S., Li Y., Kibria M.G., Hu J., Su B.L. (2021). n-p Heterojunction of TiO_2_-NiO core-shell structure for efficient hydrogen generation and lignin photoreforming. J. Colloid Interface Sci..

[18] Du X., Bai X., Xu L., Yang L., Jin P. (2020). Visible-light activation of persulfate by TiO_2_/g-C_3_N_4_ photocatalyst toward efficient degradation of micropollutants. Chem. Eng. J..

[19] Player L.C., Chan B., Turner P., Masters A.F., Maschmeyer T. (2018). Bromozincate ionic liquids in the Knoevenagel condensation reaction. Appl. Catal. B Environ..

[20] Pereira B.I., Devine O.P., Vukmanovic-Stejic M., Chambers E.S., Subramanian P., Patel N., Virasami A., Sebire N.J., Kinsler V., Valdovinos A. (2019). Senescent cells evade immune clearance via HLA-E-mediated NK and CD^8+^ T cell inhibition. Nat. Commun..

[21] Lin L., Lin P., Song J., Zhang Z., Wang X., Su W. (2023). Boosting the photocatalytic activity and stability of Cu_2_O for CO_2_ conversion by LaTiO_2_N. J. Colloid Interface Sci..

[22] Duan J., Zhao H., Zhang Z., Wang W. (2018). The Z-scheme heterojunction between TiO_2_ nanotubes and Cu_2_O nanoparticles mediated by Ag nanoparticles for enhanced photocatalytic stability and activity under visible light. Ceram. Int..

[23] Li J.M., Tsao C.W., Fang M.J., Chen C.C., Liu C.W., Hsu Y.J. (2018). TiO_2_-Au-Cu_2_O Photocathodes: Au-Mediated Z-Scheme Charge Transfer for Efficient Solar-Driven Photoelectrochemical Reduction. ACS Appl. Nano Mater..

[24] Li Z., Liu J., Wang D., Gao Y., Shen J. (2012). Cu_2_O/Cu/TiO_2_ nanotube Ohmic heterojunction arrays with enhanced photocatalytic hydrogen production activity. Int. J. Hydrogen Energy.

[25] Wang Y., Zhang Y.N., Zhao G., Tian H., Shi H., Zhou T. (2012). Design of a Novel Cu_2_O/TiO_2_/Carbon Aerogel Electrode and Its Efficient Electrosorption-Assisted Visible Light Photocatalytic Degradation of 2,4,6-Trichlorophenol. ACS Appl. Mater. Interfaces.

[26] Sinatra L., LaGrow A.P., Peng W., Kirmani A.R., Amassian A., Idriss H., Bakr O.M. (2015). A Au/Cu_2_O–TiO_2_ system for photo-catalytic hydrogen production. A pn-junction effect or a simple case of in situ reduction?. J. Catal..

[27] Trenczek-Zajac A., Banas-Gac J., Radecka M. (2021). TiO_2_@Cu_2_O n-n Type Heterostructures for Photochemistry. Materials.

[28] Messaoudi O., Elgharbi S., Bougoffa A., Mansouri M., Bardaoui A., Teka S., Manai L., Azhary A. (2020). Annealing temperature investigation on electrodeposited Cu_2_O properties. Phase Transitions.

[29] Trenczek-Zajac A., Banas-Gac J., Radecka M. (2021). TiO_2_@Cu_2_O n-n Type Heterostructures for Photochemistry. Materials.

[30] Tahir D., Tougaard S. (2012). Electronic and optical properties of Cu, CuO and Cu_2_O studied by electron spectroscopy. J. Phys. Condens. Matter.

[31] Chai Y., Chen Y., Wang B., Jiang J., Liu Y., Shen J., Wang X., Zhang Z. (2022). Sn^2+^ and Cu^2+^ Self-Codoped Cu_2_ZnSnS_4_ Nanosheets Switching from p-Type to n-Type Semiconductors for Visible-Light-Driven CO_2_ Reduction. ACS Sustainable Chem. Eng..

[32] Chai Y., Kong Y., Lin M., Lin W., Shen J., Long J., Yuan R., Dai W., Wang X., Zhang Z. (2023). Metal to non-metal sites of metallic sulfides switching products from CO to CH_4_ for photocatalytic CO_2_ reduction. Nat. Commun..

[33] Wang Y., Shang X., Shen J., Zhang Z., Wang D., Lin J., Wu J.C.S., Fu X., Wang X., Li C. (2020). Direct and indirect Z-scheme heterostructure-coupled photosystem enabling cooperation of CO_2_ reduction and H_2_O oxidation. Nat. Commun..

[34] Shi Z., Jin W., Sun Y., Li X., Mao L., Cai X., Lou Z. (2023). Interface charge separation in Cu_2_CoSnS_4_/ZnIn_2_S_4_ heterojunction for boosting photocatalytic hydrogen production. Chin. J. Struct. Chem..

[35] Guan R., Wang L., Wang D., Li K., Tan H., Chen Y., Cheng X., Zhao Z., Shang Q., Sun Z. (2022). Boosting photocatalytic hydrogen production via enhanced exciton dissociation in black phosphorus quantum Dots/TiO_2_ heterojunction. Chem. Eng. J..

[36] Li H., Wang P., Yi X., Yu H. (2020). Edge-selectively amidated graphene for boosting H_2_-evolution activity of TiO_2_ photocatalyst. Appl. Catal. B Environ..

[37] Yang W., Hou H., Yang Y., Ma G., Zhan X., Yang H., Yang W. (2022). MXene-derived anatase-TiO_2_/rutile-TiO_2_/In_2_O_3_ heterojunctions toward efficient hydrogen evolution. Colloids Surf. A Physicochem. Eng. Asp..

[38] Dong W.-W., Jia J., Wang Y., An J.-R., Yang O.-Y., Gao X.-J., Liu Y.-L., Zhao J., Li D.-S. (2022). Visible-light-driven solvent-free photocatalytic CO_2_ reduction to CO by Co-MOF/Cu_2_O heterojunction with superior selectivity. Chem. Eng. J..

[39] Zhao X., Sun L., Jin X., Xu M., Yin S., Li J., Li X., Shen D., Yan Y., Huo P. (2021). Cu media constructed Z-scheme heterojunction of UiO-66-NH_2_/Cu_2_O/Cu for enhanced photocatalytic induction of CO_2_. Appl. Surf. Sci..

[40] Tang J.Y., Guo R.T., Zhou W.G., Huang C.Y., Pan W.G. (2018). Ball-flower like NiO/g-C_3_N_4_ heterojunction for efficient visible light photocatalytic CO_2_ reduction. Appl. Catal. B Environ..

[41] Jia Y., Liu P., Wang Q., Wu Y., Cao D., Qiao Q.-A. (2021). Construction of Bi_2_S_3_-BiOBr nanosheets on TiO_2_ NTA as the effective photocatalysts: Pollutant removal, photoelectric conversion and hydrogen generation. J. Colloid Interface Sci..

[42] Su F., Wang T., Lv R., Zhang J., Zhang P., Lu J., Gong J. (2013). Dendritic Au/TiO_2_ nanorod arrays for visible-light driven photoelectrochemical water splitting. Nanoscale.

[43] Shi Y., Li L., Xu Z., Guo F., Shi W. (2023). Construction of full solar-spectrum available S-scheme heterojunction for boosted photothermal-assisted photocatalytic H_2_ production. Chem. Eng. J..

[44] Sun X., Zhang J., Luo M., Ma J., Xian T., Liu G., Yang H. (2024). Elevating photocatalytic H_2_ evolution over ZnIn2S4@Au@Cd_0.7_Zn_0.3_S multilayer nanotubes via Au-mediating H–S antibonding-orbital occupancy. Chem. Eng. J..

[45] Su K., Yuan S.X., Wu L.Y., Liu Z.L., Zhang M., Lu T.B. (2023). Nanoscale Janus Z-Scheme Heterojunction for Boosting Artificial Photosynthesis. Small.

[46] Hou L., Li S., Lin Y., Wang D., Xie T. (2016). Photogenerated charges transfer across the interface between NiO and TiO_2_ nanotube arrays for photocatalytic degradation: A surface photovoltage study. J. Colloid Interface Sci..

[47] Zhang X., Zhang L., Xie T., Wang D. (2009). Low-Temperature Synthesis and High Visible-Light-Induced Photocatalytic Activity of BiOI/TiO_2_ Heterostructures. J. Phys. Chem. C.

[48] Su X., Zhao J., Li Y., Zhu Y., Ma X., Sun F., Wang Z. (2009). Solution synthesis of Cu_2_O/TiO_2_ core-shell nanocomposites. Colloids Surf. A Physicochem. Eng. Asp..

[49] López R., Gómez R. (2012). Band-gap energy estimation from diffuse reflectance measurements on sol–gel and commercial TiO_2_: a comparative study. J. Sol. Gel Sci. Technol..

[50] Wang X., Jiang Z., Chen H., Wang K., Wang X. (2022). Photocatalytic CO_2_ reduction with water vapor to CO and CH_4_ in a recirculation reactor by Ag-Cu_2_O/TiO_2_ Z-scheme heterostructures. J. Alloys Compd..

[51] Sun X., Zhang J., Ma J., Xian T., Liu G., Yang H. (2024). Synthesis of strongly interactive FeWO_4_/BiOCl heterostructures for efficient photoreduction of CO_2_ and piezo-photodegradation of bisphenol A. Chem. Eng. J..

[52] Tian N., Huang H., Wang S., Zhang T., Du X., Zhang Y. (2020). Facet-charge-induced coupling dependent interfacial photocharge separation: A case of BiOI/g-C3N4 p-n junction. Appl. Catal. B Environ..

[53] Shao Y.Y., Yuan J.H., Li X.N., Li Z.M., Hu Y.L., Cheng Z.L., Liu R.W., Zheng R., Hou Y.D., Li M. (2023). Compositional dependence of high temperature oxidation resistance in the L_12_-strengthened high-thermostability copper alloys. Corros. Sci..

