# Supplementary material for: Effect of Cu valence states on conduction band position and reduction selectivity of TiO2-based heterojunction photocatalysts
Source: iScience. 2025 May 19;28(6):112697. doi: 10.1016/j.isci.2025.112697 (PMC12164044; doi:10.1016/j.isci.2025.112697)
Supplement: Document S1. Figures S1–S6 and Table S1 [file mmc1.pdf]

iScience, Volume *28*

Supplemental information

Effect of Cu valence states on conduction band position and reduction selectivity of TiO_2-based heterojunction photocatalysts

Hong Qian, Binxia Yuan, Yuhao Liu, Li Wang, Rui Zhu, and Pengyu Dong

Supporting information

Fig. S1 showed the XRD diagrams of the different samples obtained at 0 mmol, 2.5 mmol, 5 mmol, and 25 mmol NaOH. Without NaOH, the sample mainly exhibited diffraction peaks of anatase TiO_2 and Cu. As the amount of NaOH increased, the diffraction peaks of anatase TiO_2 gradually decreased and grown along the TiO_2 (200) crystal surface.

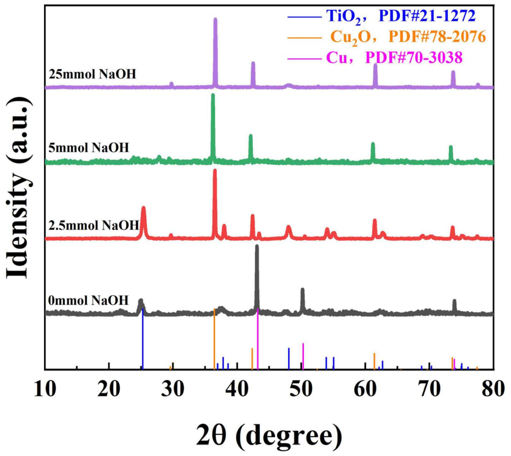

Fig. S1 XRD patterns of 0, 2.5, 5 and 25 mmol NaOH.

Fig. S2(a) and S2(b) were SEM images of 5 and 25 mmol NaOH, respectively. Fig. S2(c-g) was the EDX mapping diagram of 5 mmol NaOH. Fig. S3 showed TEM images, it can be found that the morphology of the samples developed from granular to flaky and granular mixture with the amount of NaOH increased. When the content of NaOH was 0 mmol, TiO_2 and Cu exhibited agglomerated small nanoparticles. When the content of NaOH was 2.5mmol, the sample exhibited good dispersion and uniform morphology, and presented as olive shaped nanoparticles. When the content of NaOH was greater than 5mmol , the morphology began to develop towards flaky morphology.

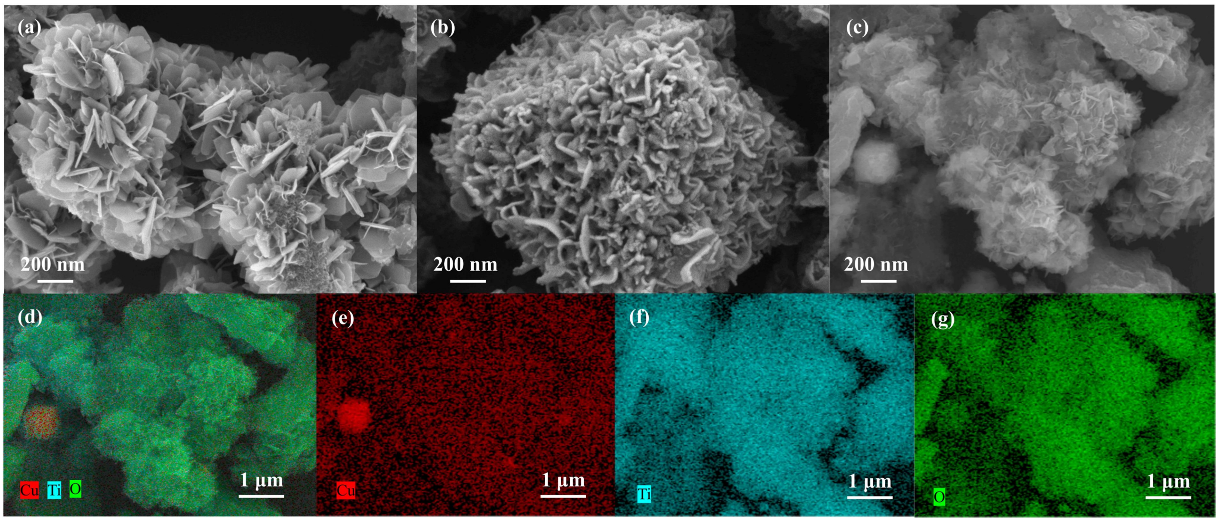

Fig. S2 SEM images of samples obtained at (a) 5 mmol NaOH; (b) 25 mmol NaOH; (c-g) EDX mapping of 5 mmol NaOH.

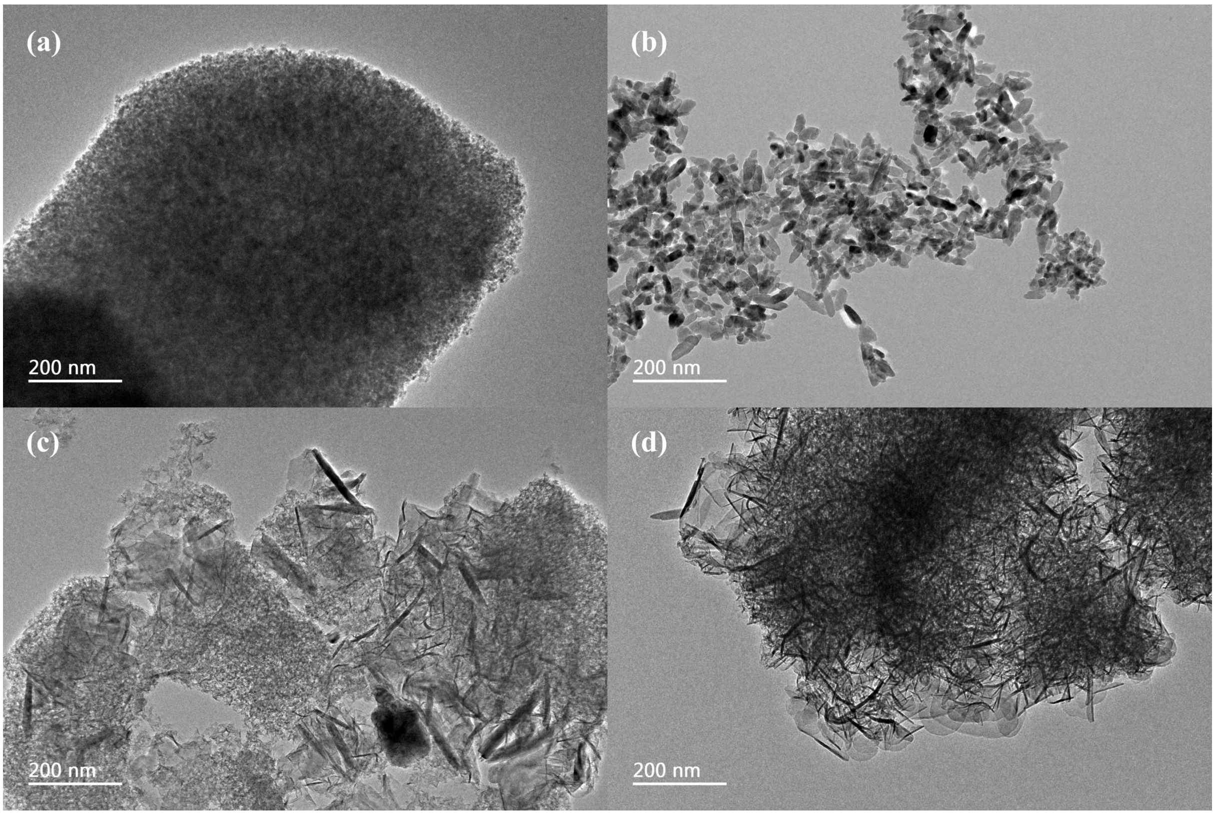

Fig. S3 TEM images of different NaOH contents: (a) 0 mmol NaOH; (b) 2.5 mmol NaOH; (c) 5 mmol NaOH; (d) 25 mmol NaOH.

Fig. S4 (a-c) showed the UV visible absorption spectrum, electrochemical impedance spectrum (EIS), and transient photocurrent curves. The absorption value and band gap of the

samples under UV and visible light increased first and then decreased, and the band edge position of the absorption value shifted significantly to red. The radius of the EIS curves first decreased and then increased, and the transient current density first increased and then decreased, indicated that the 2.5mmol NaOH sample had the best catalytic performance.

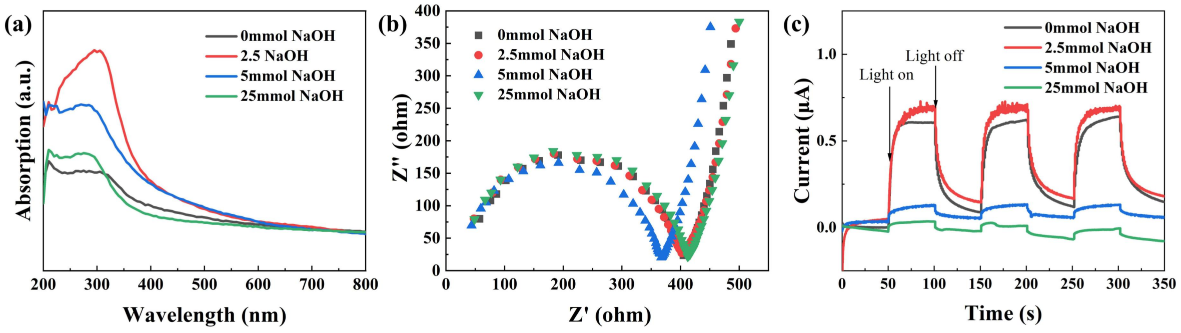

Fig. S4 (a) UV–vis absorption spectra; (b) EIS curves; (c) transient photocurrent curves.

Fig. S5 showed the band gap transition diagram and the Mott-Schottky curve. The bandgap width gradually increased, and the Mott-Schottky curve exhibited both positive and negative slopes.

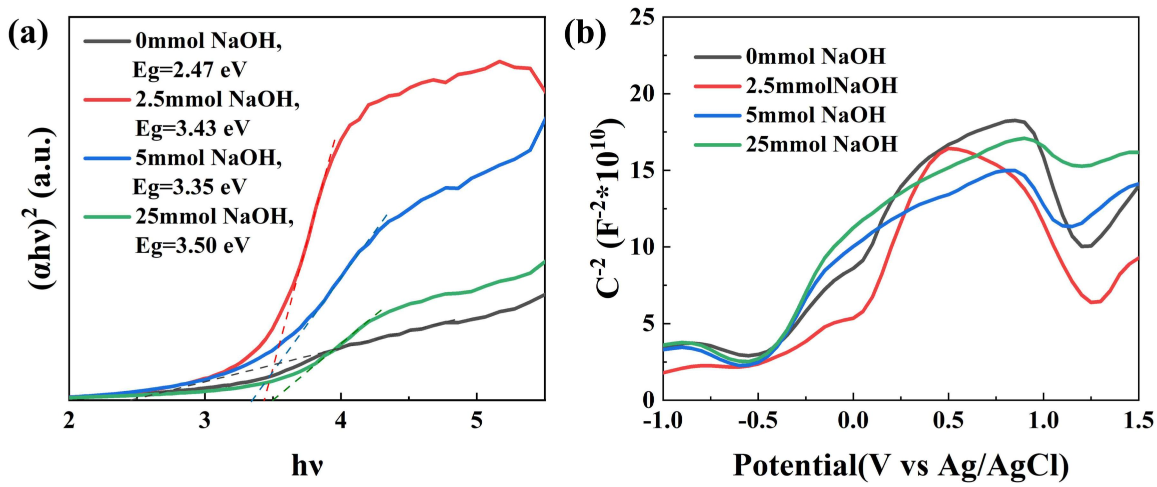

Fig. S5 (a) band gap evaluation from the plots of $(\alpha h\nu)^2$ versus $h\nu$; (b) Mott-Schottky curves.

Table S1 Multiexponential fit parameters for the decay of photoluminescence lifetime after excitation at 370 nm.

Sample	CT-0.15	CTC-1	TC-2
y_0	$0.00381 \pm 2.48248 \times 10^{-4}$	$0.00277 \pm 2.378 \times 10^{-4}$	$0.00329 \pm 2.86198 \times 10^{-4}$
A_1 [a]	0.51851 ± 0.01306	0.50468 ± 0.0093	0.54579 ± 0.00885
t_1 [b]	0.36615 ± 0.01323	0.30414 ± 0.01019	0.53508 ± 0.01519
A_2 [a]	0.3506 ± 0.01123	0.35296 ± 0.00744	0.31155 ± 0.00685
t_2 [b]	2.22242 ± 0.09225	2.5581 ± 0.08201	4.18492 ± 0.16884
A_3 [a]	0.12979 ± 0.00357	0.14055 ± 0.00322	0.14402 ± 0.00518
t_3 [b]	18.59179 ± 0.48857	20.41266 ± 0.4503	23.58081 ± 0.68924
τ_{Ave} (ns)	3.38	3.93	4.99

[a] $A_1 + A_2 + A_3 = 1$

[b] The calculation formula of average lifetime $\tau_{Ave} = \sum t_i \times A_i$.

Fig. S6 displayed the band structure and density of states (DOS) for different heterostructures. After the formation of the Cu_2O/TiO_2 heterojunction, the Cu_2O 3d orbitals facilitated the formation of defect energy levels, promoting electron transport. In comparison to the Cu_2O/TiO_2 heterojunction, the $Cu_2O/TiO_2/Cu$ heterojunction exhibited a noticeable downward shift in the band structure towards more negative energy values. This observation was consistent with the analysis conducted using Mott-Schottky measurements.

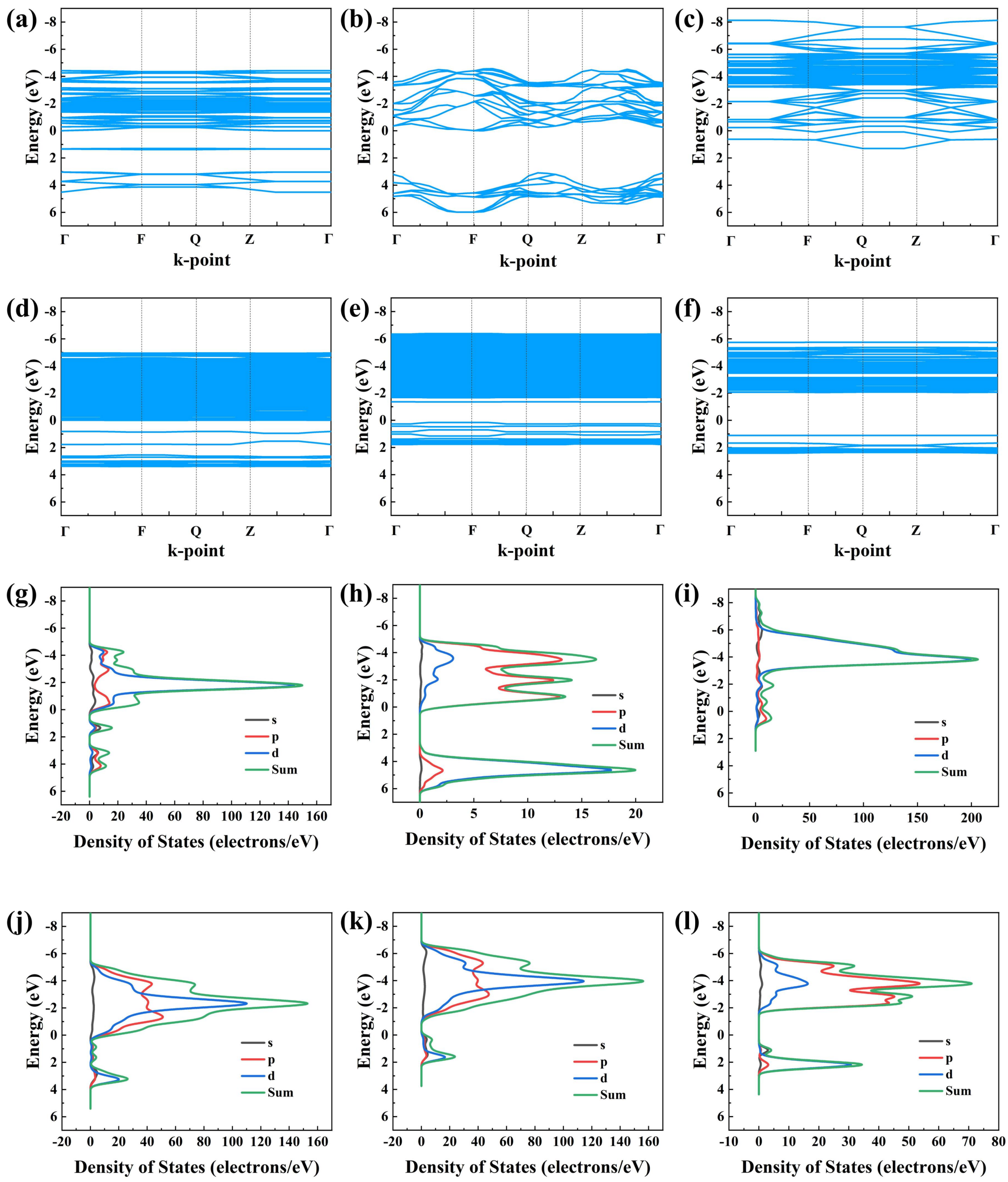

Fig. S6 Energy bands of (a) Cu_2O (111) facet, (b) TiO_2 (101) facet, (c) Cu (111) facet,(d) TiO_2 (101)/Cu_2O (111), (e) Cu_2O (111)/TiO_2 (101)/Cu, (f) TiO_2 (101)/Cu; DOS of (g) Cu_2O (111) facet, (h) TiO_2 (101) facet, (i) Cu (111) facet,(j) TiO_2 (101)/Cu_2O (111), (k) Cu_2O (111)/TiO_2 (101)/Cu, (l) TiO_2 (101)/Cu.
